# Adverse Events Post Smallpox-Vaccination: Insights from Tail Scarification Infection in Mice with *Vaccinia virus*

**DOI:** 10.1371/journal.pone.0018924

**Published:** 2011-04-15

**Authors:** Bruno E. F. Mota, Nadia Gallardo-Romero, Giliane Trindade, M. Shannon Keckler, Kevin Karem, Darin Carroll, Marco A. Campos, Leda Q. Vieira, Flávio G. da Fonseca, Paulo C. P. Ferreira, Cláudio A. Bonjardim, Inger K. Damon, Erna G. Kroon

**Affiliations:** 1 Laboratório de Vírus, Departamento de Microbiologia, Universidade Federal de Minas Gerais, Belo Horizonte, Minas Gerais, Brazil; 2 Poxvirus Program, Centers for Disease Control and Prevention, Atlanta, Georgia, United States of America; 3 Centro de Pesquisas René Rachou, FIOCRUZ, Belo Horizonte, Minas Gerais, Brazil; 4 Departamento de Bioquímica e Imunologia, Universidade Federal de Minas Gerais, Belo Horizonte, Minas Gerais, Brazil; 5 Laboratório de Virologia Comparada, Departamento de Microbiologia, Universidade Federal de Minas Gerais, Belo Horizonte, Minas Gerais, Brazil; Indian Institute of Science, India

## Abstract

Adverse events upon smallpox vaccination with fully-replicative strains of *Vaccinia virus* (VACV) comprise an array of clinical manifestations that occur primarily in immunocompromised patients leading to significant host morbidity/mortality. The expansion of immune-suppressed populations and the possible release of *Variola virus* as a bioterrorist act have given rise to concerns over vaccination complications should more widespread vaccination be reinitiated. Our goal was to evaluate the components of the host immune system that are sufficient to prevent morbidity/mortality in a murine model of tail scarification, which mimics immunological and clinical features of smallpox vaccination in humans. Infection of C57BL/6 wild-type mice led to a strictly localized infection, with complete viral clearance by day 28 p.i. On the other hand, infection of T and B-cell deficient mice (*Rag1* ^−/−^) produced a severe disease, with uncontrolled viral replication at the inoculation site and dissemination to internal organs. Infection of B-cell deficient animals (µMT) produced no mortality. However, viral clearance in µMT animals was delayed compared to WT animals, with detectable viral titers in tail and internal organs late in infection. Treatment of *Rag1* ^−/−^ with rabbit hyperimmune anti-vaccinia serum had a subtle effect on the morbidity/mortality of this strain, but it was effective in reduce viral titers in ovaries. Finally, NUDE athymic mice showed a similar outcome of infection as *Rag1* ^−/−^, and passive transfer of WT T cells to *Rag1* ^−/−^ animals proved fully effective in preventing morbidity/mortality. These results strongly suggest that both T and B cells are important in the immune response to primary VACV infection in mice, and that T-cells are required to control the infection at the inoculation site and providing help for B-cells to produce antibodies, which help to prevent viral dissemination. These insights might prove helpful to better identify individuals with higher risk of complications after infection with poxvirus.

## Introduction

The *Vaccinia virus* (VACV) is a member of *Poxviridae* family and the *Orthopoxvirus* genus, which also includes several human pathogens such as *Variola virus* (VARV), *Cowpox virus* (CPXV), *Monkeypox virus* (MPXV), and the mouse-specific pathogen *Ectromelia virus* (ECTV)[1]. The use of live, replicative VACV was effective in providing protection against smallpox through the elicitation of strong acute immune responses, followed by viral replication control and induction of immune memory [2]. However, in patients with immunodeficiency, virus replication is poorly controlled and life threatening adverse events have been reported [3]. Despite numerous recent publications on vaccine-elicited immunity [4], [5], [6], [7], little progress has been made in understanding vaccination-related adverse events. It is difficult to extrapolate this information from current animal model studies, as most studies try to model systemic orthopoxvirus disease in humans and therefore have used routes of administration that produce a systemic infection in immunocompetent mice, such as intranasal and intraperitoneal routes with high virus inoculation [6], [8]. Vaccination in humans however, is performed by inoculating the virus via multiple skin punctures that produce a localized infection, without systemic involvement in immunocompetent patients (although traces of blood borne viral DNA have been seen in a minority of patients in a few studies), but can cause a wide range of complications in immunocompromised individuals [9]. Tail scarification (ts) infection in mice provides a useful model to study the complications derived from VACV inoculation, since it resembles the immunological and virological parameters of human smallpox vaccination [10].

Studies using localized poxvirus infection showed that the generation of a Th1 response is important for the control of these infections. Freyschmidt and colleagues reported that upon VACV infection by tail scarification, Relb knockout mice (deficient in a transcription factor belonging to the NF-κB family) have a more severe disease than WT mice and that this higher susceptibility is related to their inability to mount a normal Th1 response [11]. Additionally, *in vivo* overexpression of the Th1 cytokine IL-1α (K14/IL-1 α) differentially modulates the immune response to VACV, leading to a higher control of viral replication when compared to wild type mice [12]. Furthermore, a recent study has shown that upregulation of interleukin (IL) 17 in a mouse model of atopic dermatitis decrease NK cell activity and led to a higher viral replication at the skin [13]. Regarding human studies, Howell and co-workers reported that human skin biopsies from patients with atopic dermatitis (a Th2 inflammatory skin disease) have a defective innate immune response to VACV [14]. However, due to the diverse attributes of a “Th1 response”, it is not known exactly which facet of this response is crucial to prevent VACV dissemination.

Among the main effectors elicited by the Th1 response, T and B cells are the better studied. Their participation in the immune response to a primary poxvirus infection has been well-documented in several models of poxvirus infection. Upon intraperitoneal VACV infection, a strong humoral immune response is necessary to control the infection and CD8^+^ T cell response is needed only when the antibody response is abrogated [15]. In the case of the mouse-specific pathogen ECTV infection, both T and B cells are necessary to control the infection and to avoid mortality [8], [16]. The role of humoral immunity has been revealed also by the prophylactic or therapeutic use of anti VACV antibodies in mice [17].

Despite the generation of a significant amount of data focusing on the understanding of primary VACV infection immunity by many research groups, no definitive conclusions about which component of the host immune system is crucial to an effective immune response to poxvirus inoculations in human vaccinees have yet been presented. While some authors pointed to T cells as the main cell type responsible for viral clearance based on animal studies and clinical trials [3], [11], others believed instead that the antibodies are essential to this process [18]. Hence, the objective of the present study is to add some insights into the understanding of the relative role of T cells, B cells and pro-inflammatory cytokines in the response to a localized, primary poxvirus infection. Using an array of knockout mice, we saw that the deletion of pro-inflammatory cytokines (IL-12/23, TNFα or IFNγ) or immune mediators (iNOS) had no effect on the immune response to VACV and that T and B cells are required for VACV clearance, with a more important role played by T cells.

## Results

### Absence of single pro-inflammatory mediators does not affect the outcome of infection

**Figure 1:**
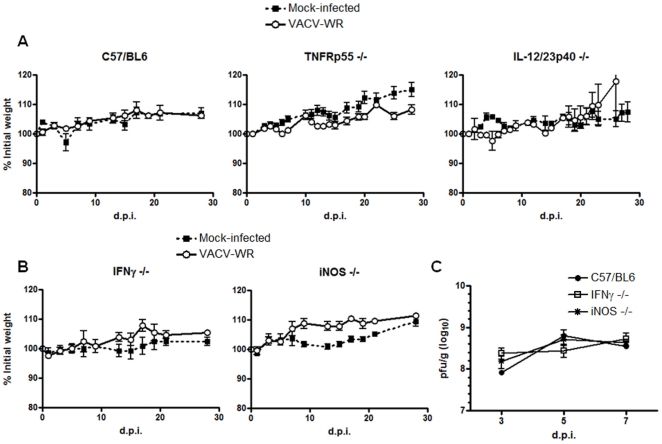
VACV infection in single-cytokine deficient mice did not alter the outcome of infection. C57BL/6 (*n* = 6), IL-12/23^−/−^ (*n* = 4), TNFR^−/−^ mice (*n* = 7), IFNγ^−/−^ (*n* = 6) and iNOS^−/−^ (*n* = 6) animals were infected with 10^7^ p.f.u. of VACV-WR by tail scarification. (**A**) Weight curve of C57BL/6, IL-12/23^−/−^, TNFR^−/−^ mice. (**B**) Weight curve of IFNγ^−/−^ and iNOS^−/−^ mice. (**C**) WT, IFNγ^−/−^ and iNOS^−/−^ animals were euthanized at days 3, 5 and 7 post-infection (p.i.) and viral titers in tails were assessed by titration in BSC-40 cells. Bars represent the mean and standard error (SEM). The results shown are representative of two separate experiments.

To test the role of inflammatory cytokines in this model, animals deficient in IL-12/23 or TNF were infected with VACV-WR by tail scarification. Like the wild type mice (C57BL/6), these strains did not show any weight loss or signs of disseminated disease (**Figure 1A**). Furthermore, IFNγ and iNOS deficient mice also did not show signs of disseminated disease (**Figure 1B**). Nevertheless, IFNγ^−/−^ mice showed different lesion development kinetics, with a delayed healing starting at day 10 p.i. and continuing throughout the experiment (until day 28 p.i.). At day 10 p.i., WT animals developed a dry scab compared to a more purulent lesion in IFN-γ-deficient mice (**Figure S1**). iNOS ^−/−^ mice showed the same lesion kinetics as WT mice (**Figure S1**), suggesting that the phenotype in IFN-γ-deficient mice was not related to lack of induction of iNOS. In spite of that, viral titers in tails were similar among WT, iNOS ^−/−^ and IFNγ-deficient mice at days 3, 5 and 7 p.i. (**Figure 1C**), suggesting-that these cytokines/immune mediator have a lesser impact on the VACV infection control in the presented model.

### Mice lacking adaptive immunity had severe disease with higher viral replication in organs

**Figure 2:**
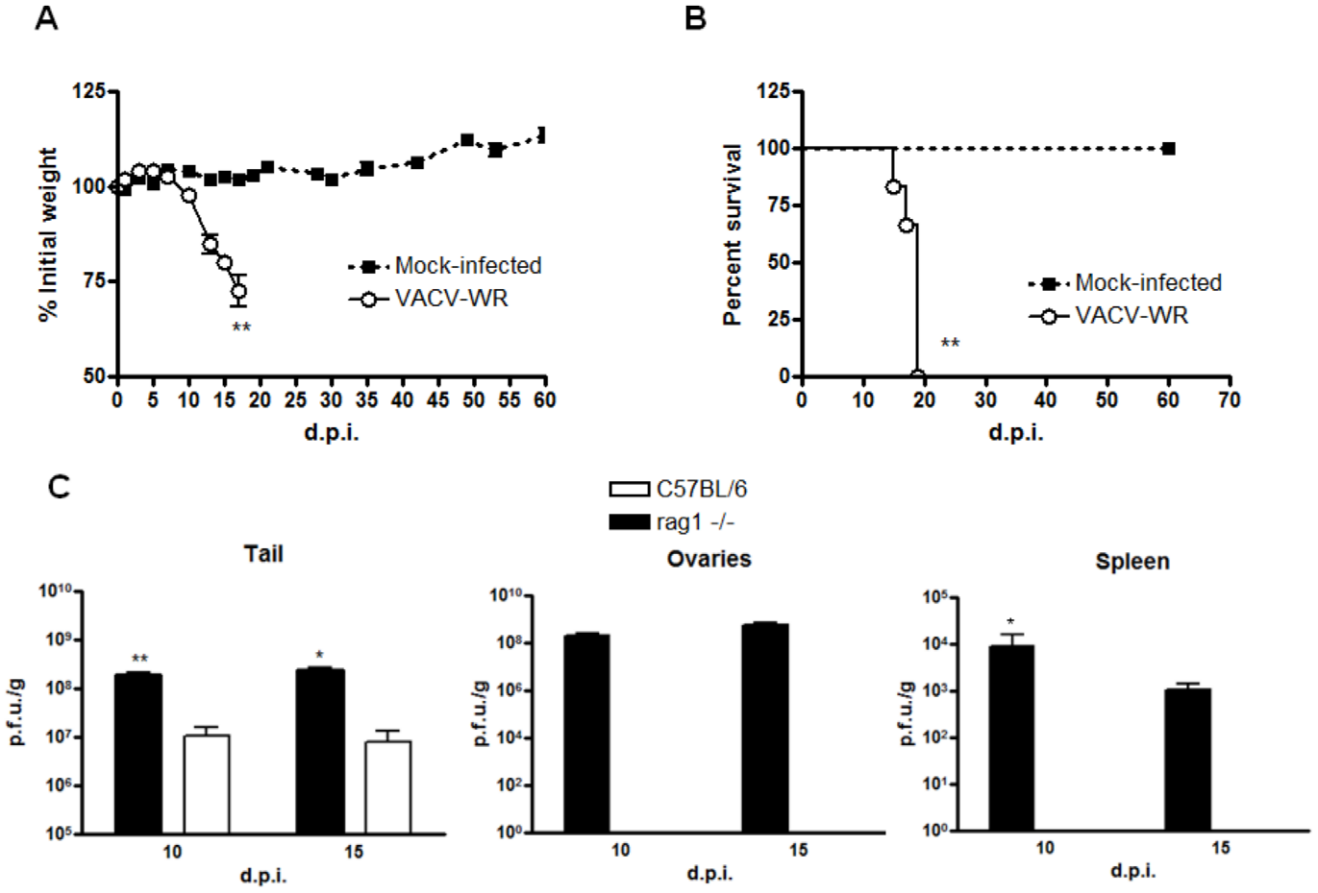
T and B-cell deficient mice had severe disease upon tail scarification infection with VACV-WR. *Rag1* ^−/−^ animals (n = 6) were infected with 10^7^ p.f.u. of VACV-WR by tail scarification. (**A**) Weight curve of infected or mock-infected *Rag1* ^−/−^ mice (**p<0.01, unpaired t test). (**B**) Animals were followed during 60 days post-infection (p.i.) to compute mean survival (**p<0.01, log-rank test). (**C**) WT and *Rag1* ^−/−^ animals were euthanized at the times indicated and the tail, spleen and ovaries were removed and titrated in BSC-40 cells. Bars represent the mean and standard error (SEM). The results are representative of three experiments.

**Figure 3:**
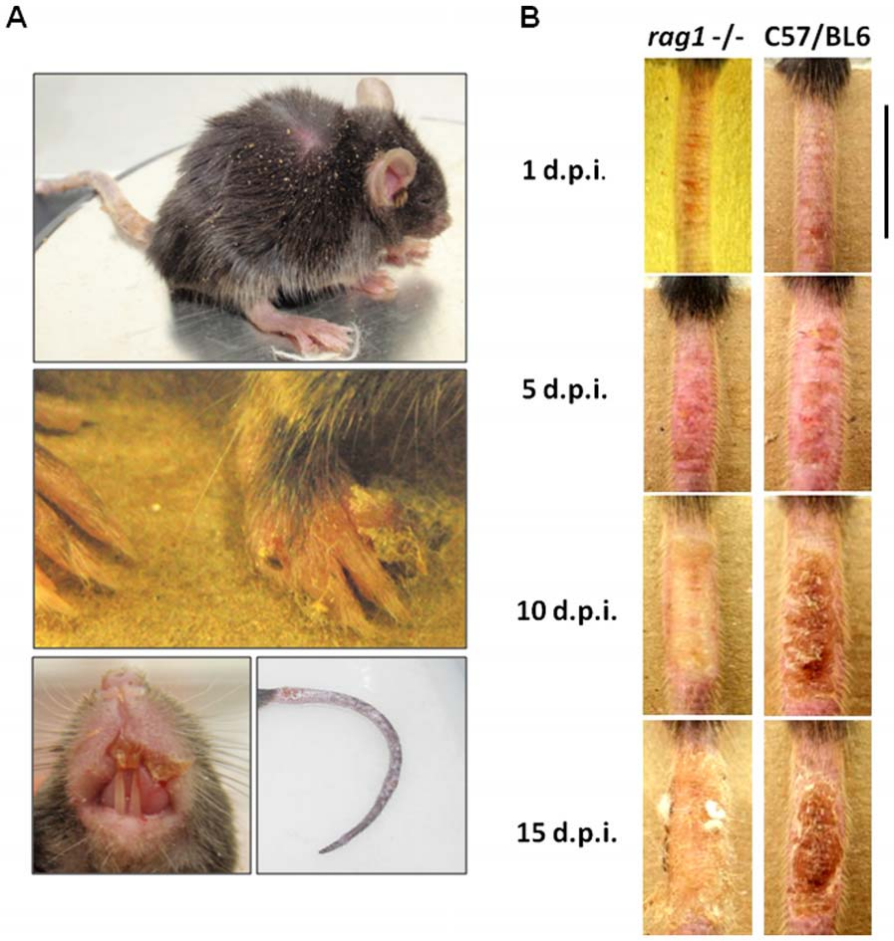
Clinical signs and lesion kinetic in T and B-cell deficient mice. *Rag1* ^−/−^ animals (n = 6) were infected with 10^7^ p.f.u. of VACV-WR by tail scarification. (**A**) Clinical signs of disseminated disease in *Rag1* ^−/−^. Starting on the second week post-infection, *Rag1* ^−/−^ mice had signs of generalized disease, such as ruffled fur, arched back and disseminated lesions, primarily at the face, forepaw and tail. The animal shown is representative of the group. (**B**) *Rag1* ^−/−^ presented a delayed lesion healing compared to WT mice. Note the ulcerative, purulent nature of the lesion in *Rag1* ^−/−^ compared to the scab formed in C57BL/6 animals. Photos are representative of the groups indicated. Black bar represents 1 cm.

Mice lacking T and B cells (*Rag1* ^−/−^) were also inoculated with VACV-WR by tail scarification. Unlike wild type and single cytokine-deficient animals, *Rag1* ^−/−^ mice had a statistically significant weight loss starting at the second week post-infection (one-way ANOVA, *p*<0.01; **Figure 2A**) and 100% of them reached the euthanasia end-point (log-rank test *p*<0.01, median survival 17 days; **Figure 2B**). Moreover, beginning at the second week of infection, all the *Rag1* ^−/−^ mice clearly showed clinical signs of disseminated disease, such as ruffling fur, arched back and disseminated poxvirus lesions, primarily on the face, forepaw and tail (**Figure 3A**).

The lesions at the inoculation site showed quite different kinetics in *Rag1* ^−/−^ mice when compared to WT mice. While in the latter the lesion progressed from papules/pustules to a scab in the first 10 days of infection, in the former the lesion took longer to heal, with no complete healing by day 15 p.i. (**Figure 3B**). This finding leads us to evaluate the viral replication at this site. Indeed, viral replication was higher at the initial site of infection in *Rag1* ^−/−^ mice (t test, *p*<0.01). Additionally, while WT mice presented a strictly localized infection with no viral replication in evaluated internal organs, *Rag1* ^−/−^ had an extensive replication in spleen and ovaries at both analyzed time points (*p*<0.05, one sample t test, **Figure 2C**).

### Antibody response protects mice against viral dissemination, but not from mortality

**Figure 4:**
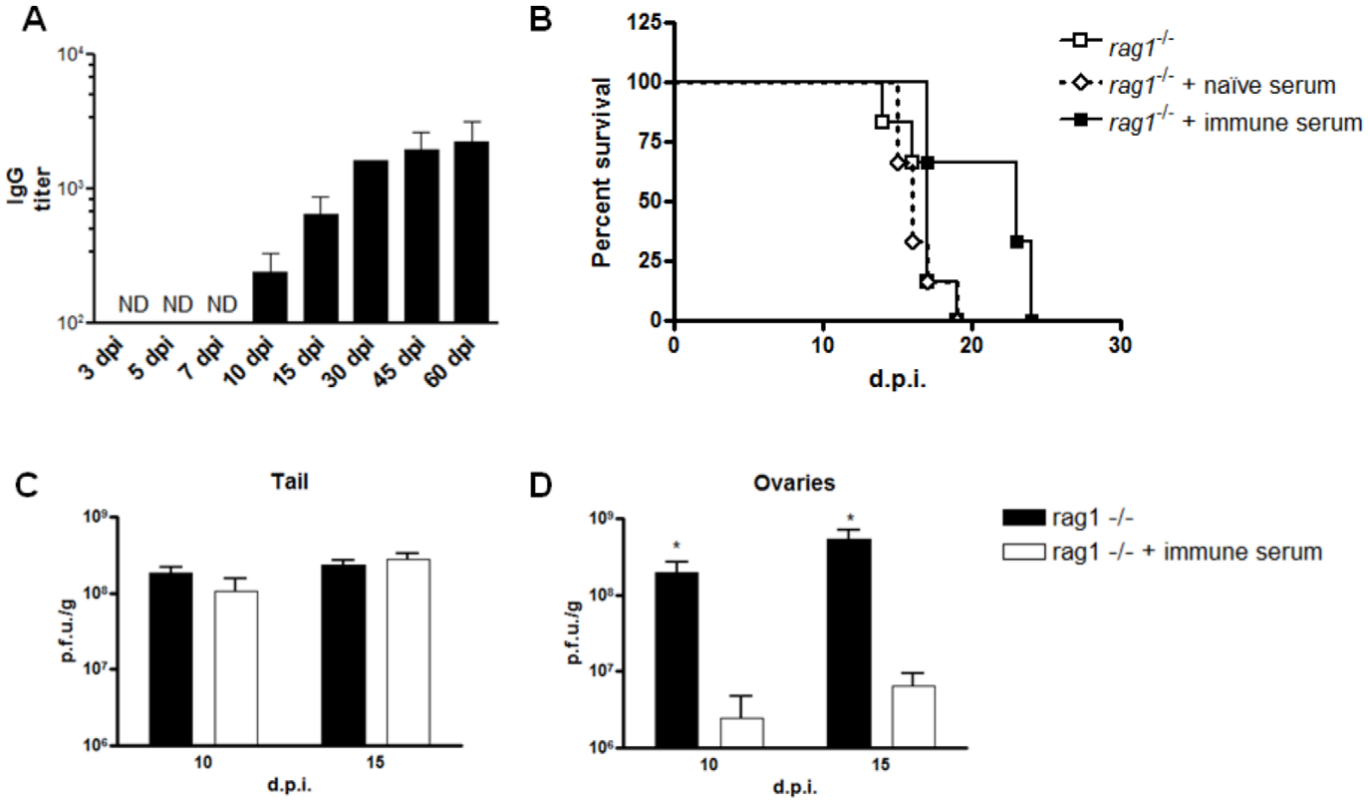
Antibody response seems not crucial to prevent mortality, but it is important to block viral dissemination. (**A**) WT (C57BL/6) mice were infected with 10^7^ p.f.u. of VACV-WR by tail scarification and euthanized as described. Sera were collected at the times indicated. Indirect IgG ELISA was performed using purified VACV-WR as antigen (10^6^ p.f.u./well). Bars represent mean and standard deviation (SD). ND: not detected. (**B**) Survival curve of *Rag1* ^−/−^ animals inoculated with anti-VACV rabbit serum or rabbit naïve serum intraperitoneally every week after infection. At days 10 and 15 p.i., the tails (**C**) and the ovaries (**D**) were removed and titrated in BSC-40 cells. Bars represent mean and standard error (SEM). *p<0.05, unpaired t test.

Analysis of the antibody response in wild type mice (C57BL/6) showed that ELISA IGg titers were detectable starting at day 10 p.i. and increased until day 60 p.i., the last day analyzed (**Figure 4A**). The appearance of IgG correlates well with the time *Rag1* ^−/−^ animals started to lose weight (**Figure 2A**). Hence, we evaluate if antibody treatment could prevent the morbidity/mortality seen in *Rag1* ^−/−^ mice. Animals treated with rabbit anti-vaccinia serum had a delayed mortality compared to non-treated animals (**Figure 4B**), even though the difference was not statistically significant. When the viral replication was analyzed at days 10 and 15 p.i. in antibody-treated mice, viral levels were similar in the tail (**Figure 4C**), but otherwise statistically smaller in the ovaries of treated animals (**Figure 4D**) (t test, *p*<0.05).

**Figure 5:**
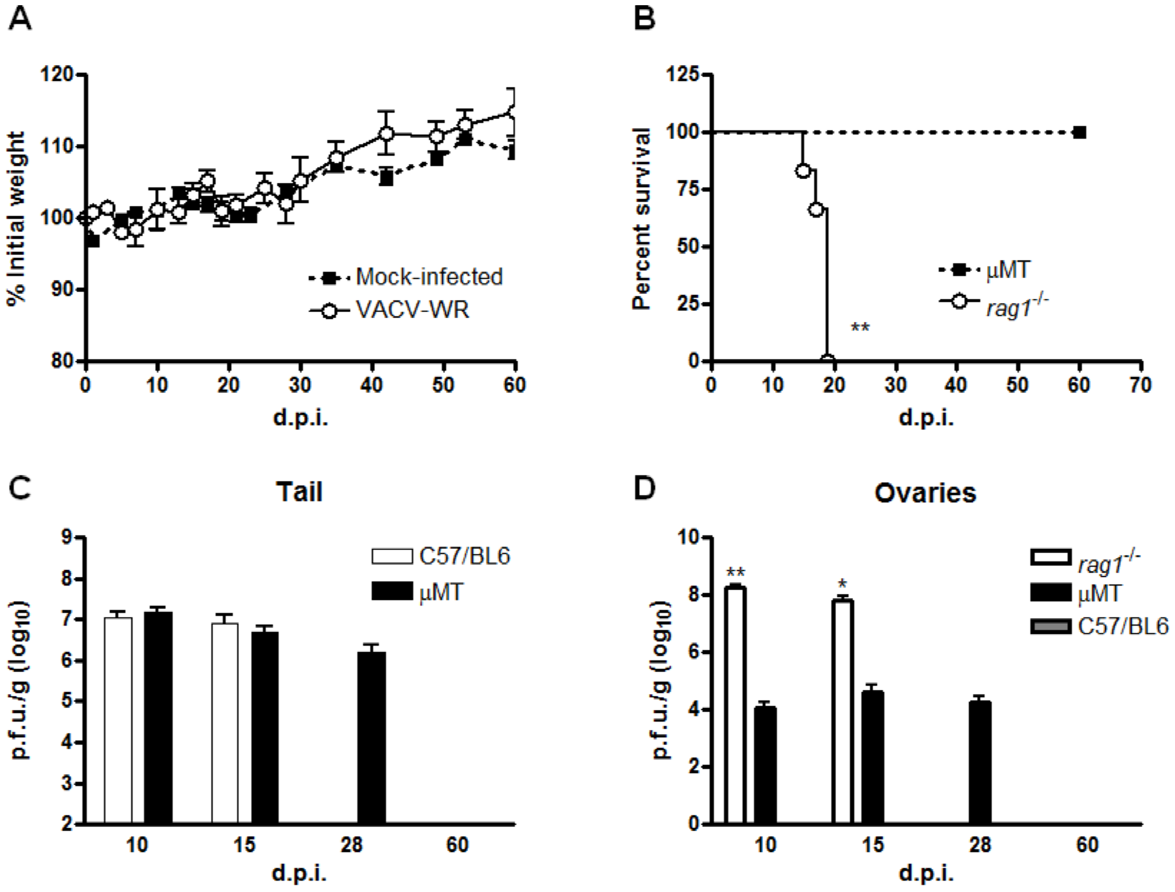
B-cell deficient mice did not show any signs of disseminated disease, but had delayed viral clearance. µMT mice (*n* = 6) were infected with 10^7^ p.f.u. of VACV-WR by tail scarification. (**A**) Weight curve of µMT mice infected with VACV-WR or mock-infected. (**B**) The same animals were followed for 60 days to compute mean survival. (**C**) Viral titers were determined in tails of C57BL/6 and µMT mice at days 10, 15, 28 and 60 p.i. (*p<0.05 and **p<0.01, t test). (**D**) Viral titers were determined in ovaries of µMT mice at days 10, 15, 28 and 60 p.i. and of *Rag1* ^−/−^ mice at days 10 and 15 p.i.(*p<0.05 and **p<0.01, t test). Bars represent mean and standard error (SEM). Results shown are representative of two experiments.

To test the role of antibodies with another approach, B cell-deficient mice (µMT) were infected with VACV-WR and followed as above. These animals did not show any weight loss in the time analyzed (**Figure 5A**) and no mortality was observed (**Figure 5B**). Unexpectedly, these mice had a delayed control of viral replication at the initial site of infection, since they had detectable viral particles in the tail by day 28 p.i., while WT animals had completely cleared the infection (**Figure 5C**). Furthermore, the animals deficient only in B cells had higher titers on internal organs, such as the ovaries, than the WT mice, but smaller than the B and T-cell deficient mice (**Figure 5D**). By day 60 p.i., all µMT mice had completely cleared the infection (**Figures 5C and 5D**).

### Animals deficient in T cells had a similar outcome of infection as Rag^−/−^ mice

**Figure 6:**
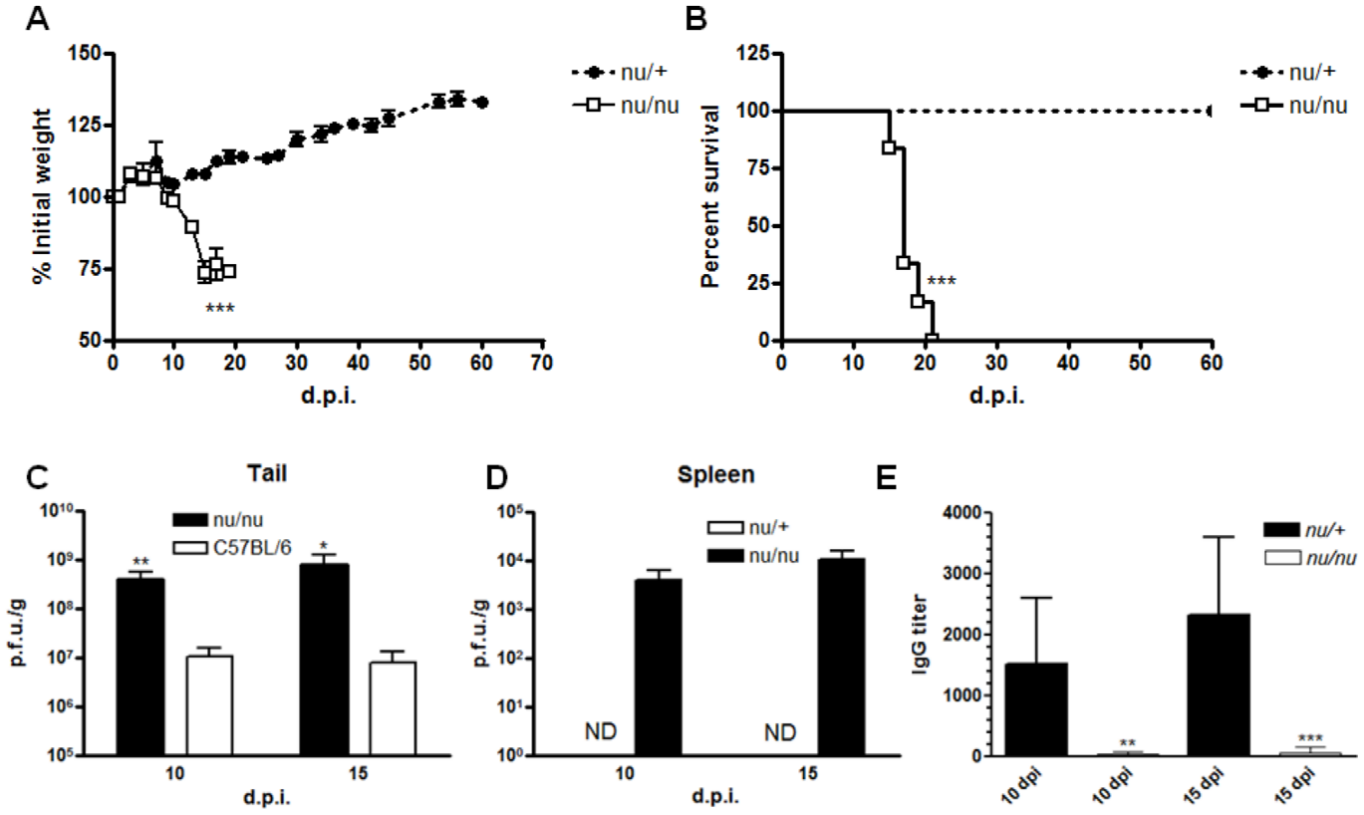
Mice lacking T cells are as susceptible to primary VACV infection as *Rag1* ^−/−^ mice. NUDE athymic mice (*nu/nu*) and their wild type littermates (*nu/+*) were infected with 10^7^ p.f.u. of VACV-WR by tail scarification. (**A**) Weight curve of NUDE mice (***p<0.001, unpaired t test). (**B**) Survival curve of *nu/nu* and *nu/+* after infection (***p<0.001, log-rank test). (**C**) Viral titers were determined in tail of C57BL/6 and *nu/nu* mice (**p<0.01 day 10 p.i. and *p<0.05, t test). Data of viral titer in tails of *nu/+* mice are missing. (**D**) Spleens of *nu/nu* and *nu/+* mice were removed at the times indicated and virus titer was determined in BSC-40 cells. (**E**) VACV-specific IgG antibodies were determined by ELISA in serum of *nu/nu* and *nu/+* mice (**p<0.01 and ***p<0.001, t test). Bars represent the mean and standard error (SEM), except for 6E, where bars represent mean and standard deviation (SD). The results shown are representative of two experiments. ND: not detected.

As the antibody response seems not to be sufficient to control morbidity/mortality in VACV primary infection, we sought to evaluate the role of T cells in viral clearance. NUDE athymic animals (*nu/nu*) or their wild type counterparts (*nu/+*) were infected with VACV by tail scarification and followed as before. NUDE mice (in opposition to their *nu/+* littermates) showed similar clinical signs of infection to *Rag1* ^−/−^ mice, including decreased activities, disseminated lesions in the tail (data not shown) and significant weight loss (*p*<0.001, unpaired t test, **Figure 6A**), culminating with the death of all animals in the group (Mean survival: 17 days, *p*<0.001 compared to *nu/+* animals, **Figure 6B**). Again, as in *Rag1* ^−/−^ mice, clinical signs of disease in *nu/nu* animals correlate with a higher viral replication in the initial site of infection (t test, *p*<0.05, **Figure 6C**) and in the spleen at days 10 and 15 p.i. (**Figure 6D**). Consistent with the role of T cells to provide help to B cells, the ELISA IgG titer in sera of NUDE animals was significantly reduced when compared to *nu/+* animals (unpaired t test, *p*<0.01 day 10 p.i. and *p*<0.001 day 15 p.i., **Figure 6E**).

### Concomitant passive transfer of CD4^+^ and CD8^+^ T cells, but not of these cells alone, fully protects mice against morbidity and mortality caused by primary VACV infection

**Figure 7:**
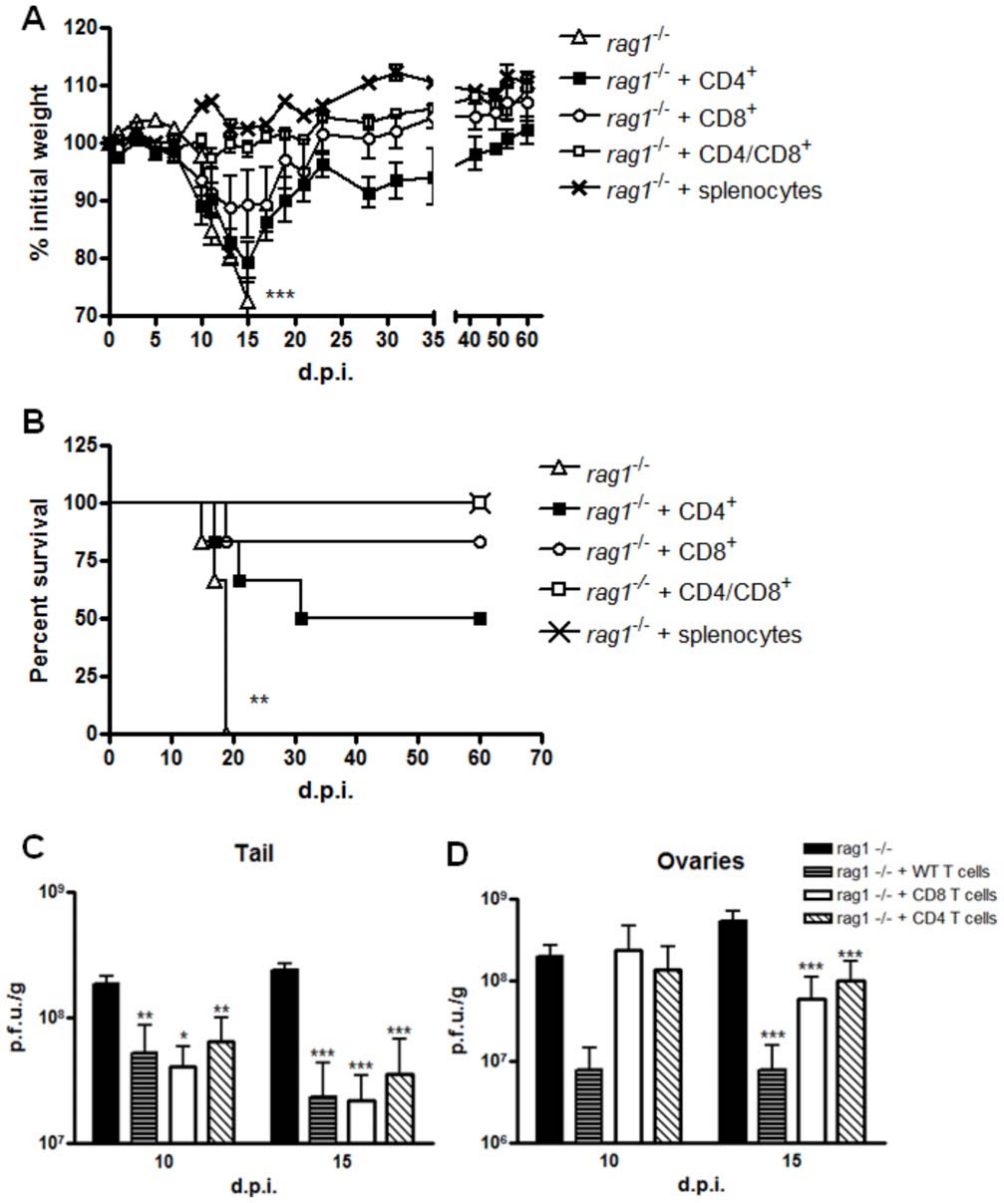
*Rag1* ^−/−^ mice passively transferred with both T cells were fully protected from VACV infection. Spleens of naïve C57BL/6 mice were removed and made into single-cell suspension and this population was enriched in CD4^+^ and CD8^+^ T cells using a negative selection system. (**A**) *Rag1* ^−/−^ were inoculated intravenously with either CD4^+^ and CD8^+^ T cells or either type alone (*n* = 6 per group). As a control, *Rag1* ^−/−^ mice received splenocytes prior to isolation intravenously (10^7^ viable cells). Four days after inoculation, all animals were infected with VACV-WR by tail scarification and weighted daily (***p<0.001, paired t test). Bars represent mean and SEM. (**B**) Survival curve of *Rag1* ^−/−^ passively transferred with both CD4^+^ and CD8^+^ T cells or with either type alone, compared to the mortality in the same strain without previous treatment (**p<0.01, log-rank test). Viral titers in tail (**C**) and ovaries (**D**) of mice passively transferred with WT T cells and infected with VACV-WR. Bars represent mean and standard error (SEM) (*p<0.05, **p<0.01 and ***p<0.001 compared to *Rag1* ^−/−^ non-treated group, t test). The results shown are representative of two experiments (CD4^+^ plus CD8^+^ T cells group). For animals that received only CD4^+^ or CD8^+^ T cells, only one experiment was performed.

To further investigate the role of T cells to protect animals of generalized disease, *Rag1* ^−/−^ mice were passively transferred with populations enriched in both CD4^+^ and CD8^+^ T cells, isolated from spleens of WT mice. The purity of these enriched populations was assured by flow cytometry (CD4+ T cells: 90.2% pure; CD8^+^ T cells: 84.0% pure, **Figure S3A**). Animals that received both T cell types showed a remarkable improvement in their clinical signs, exhibiting no significant weight loss (**Figure 7A**) nor other signs of systemic infection (data not shown), similar to animals that passively received whole splenocytes (**Figure 7A**). On the other hand, animals that received CD4^+^ or CD8^+^ T cells alone showed clear weight loss and signs of disease between days 10 and 15 p.i. (*p*<0.001, **Figure 7A**), culminating with the death of 3 out of 6 animals (CD4^+^ only group) and 1 out of 6 (CD8^+^ only group) (**Figure 7B**). The lesion development kinetics of *Rag1* ^−/−^ mice reconstituted with WT T cells was similar to that of mice reconstituted with whole splenocytes, with the formation of a scab already by day 10 p.i. On the other hand, animals that received just one T cell type showed poor lesion healing by then, presenting an ulcerative lesion similar to non-treated animals (**Figure S2**). Despite the clinical signs showed by mice that received only one cell type, viral titers in the tail was diminished in these mice as well in mice that received both T cells together, compared to non-treated *Rag1* ^−/−^ animals (*p*<0.05 at day 10 p.i. and *p*<0.001 at day 15 p.i., **Figure 7C**). Furthermore, viral titer in ovaries was also diminished in all reconstituted mice, especially in mice reconstituted with WT T cells together (*p*<0.001, **Figure 7D**). To confirm the functionality of the transferred cells, flow cytometric analysis was performed with splenocytes of reconstituted mice at days 10 and 15 p.i. This analysis showed that the percentage of activated splenocytes (producing IFN-γ) is similar between mice that received both T cell types compared to animals that received only one type (Increase in the percentage of splenocytes producing IFN-γ between days 10 and 15. Mock infected: 1.28-fold increase; CD8 plus CD4: 12.8-fold increase; CD8 only: 15.1-fold increase and CD4 only: 14.66-fold increase, **Figure S3B and C**).

## Discussion

The adverse events following smallpox vaccination includes a variety of clinical manifestation, ranging from a limited viral spread around the site of inoculation to more severe outcomes, such as viral dissemination to internal organs [3], [9]. At the time of the *Variola virus* (VARV) WHO eradication campaign, these complications were relatively rare, and considering that smallpox was endemic or epidemic in these regions, smallpox immunizations were more beneficial than harmful [2]. However, with the increase of the number of immunocompromised individuals (immunosuppressive therapy, HIV/AIDS) seen today, the risk of complications upon smallpox vaccination, with fully-replicative VACV strains, is higher and may preclude widespread use in a vaccination campaign, or use as a vector against several pathogens [19]. Moreover, these immunodeficient patients are also more susceptible to infections with other zoonotic poxviruses, such as *Monkeypox virus* (MPXV) and *Cowpox virus* (CPXV) [20], [21]. Nowadays, there is an ongoing debate about which components of the host immune system are required and sufficient to control a VACV primary infection, regardless if it is natural or by means of vaccination. Some authors attribute the main role to T cells [3], [8], [11] and others to B cells and antibodies [15], [18]. Hence, our objective was to better understand those adverse events upon smallpox vaccination using the murine model of tail scarification.

Our results suggest that the infection in C57BL/6 WT mice is strictly localized, since we were not able to detect virus in internal organs (ovaries and spleen) of these mice in all times analyzed (**Figure 2C**), and no signs of weight loss was observed (**Figure 1A** and not shown), confirming other findings in the literature [11], [22]. Moreover, freshly isolated splenocytes from WT mice showed low levels of IFN-γ producing cells at days 10 and 15 p.i., suggesting that the infection is cleared in the inoculation site, with low systemic immune activation (data not shown). Many cytokines are involved in VACV clearance in other models of infection and studies in humans including IL-12 and IL-23 [23], [24], TNF-α [25] and IFN-γ [26]. Furthermore, the inducible nitric oxide synthase (iNOS) has been involved in the response to VACV *in vitro* [27], [28], but its role *in vivo* is uncertain [29]. Thus, we sought to evaluate the role of these cytokines and immune mediators in the VACV tail scarification model. IL12/23p40, TNFRp55, IFNγ and iNOS knockout animals did not show any weight loss upon infection with VACV (**Figure 1A**). Furthermore, despite the phenotypic difference in lesion kinetics, viral replication was similar in tails of WT, IFNγ KO and iNOS KO **(Figure S1 and 1C)**. These findings suggest that these cytokines alone are not important for the control of primary localized infection in mice. This could be explained by the functional redundancy of cytokines, where a single cytokine can be functionally replaced by others. For example, IL-18 is functionally similar to IL-12 [30] and is also important in VACV immune response [23]. The antiviral cytokine IL-15 is related to IL-2 and has its role in the control of VACV infection [31]. Confirming the involvement of multiple cytokines in this model, RelB (a transcription factor of the NF-κB family) knockout mice are more susceptible to VACV infection by tail scarification due to the lower IFN-γ production allied to an increase of the Th2 cytokines IL-4, IL-5 and IL-10 [11].

Given that canonical Th1 cytokines seem not to be crucial alone in the immune response to VACV, we sought to evaluate the role of adaptive immunity in this context. *Rag1* ^−/−^ animals are T and B cell deficient, due to the disruption of the recombination activation gene 1, responsible for the somatic recombination that occurred in T and B cells precursors upon maturation. Despite the lack of T and B cells, they retain normal or even higher levels of other bone marrow-derived cells, such as macrophages and natural killers (NK cells) [32]. Infection of *Rag1* ^−/−^ mice led to the development of a generalized disease, with disseminated poxvirus lesions in the face, forepaw and tail, which culminate with the mortality of 100% of the animals (**Figure 3A**). Moreover, the lesions in *Rag1* ^−/−^ mice took longer to show signs of healing when compared to WT controls and viral titers in tail of these mice were 1 log higher when compared to WT control mice (**Figure 2C**). Nevertheless, whilst WT mice had no detectable viral titers in spleen and ovaries in all times analyzed, *Rag* ^−/−^ animals harbored an intensive viral replication in these organs (**Figure 2C**). Altogether, these findings suggested that the host adaptive immunity is essential to control the replication and dissemination of VACV in the murine model of tail scarification. The role of the adaptive immunity had been appreciated in several other studies of poxvirus infection in mice [6], [8].

Even though the importance of adaptive immunity to control poxvirus infections has been universally accepted, the relative contribution of T and B cells in this process is still a matter of debate [3], [18]. Once the antibody response is essential in primary and secondary poxvirus infections using generalized infection models, such as intranasal and intraperitoneal [4], [8], [16], [33], we evaluated the role of B cells and antibodies in the present model. WT animals infected with VACV by tail scarification mounted a robust IgG antibody response. The time of appearance of long-lived IgG antibodies correlates well with the appearance of clinical signs in *Rag1* ^−/−^ mice (**Figure 2** **and** **3**), suggesting that antibodies might be relevant in this model. To test this hypothesis, *Rag1* ^−/−^ mice were infected with VACV-WR and passively immunized with polyclonal rabbit anti-VACV serum. Treated animals showed a slightly improvement in their mortality compared to *Rag1* ^−/−^animals treated with rabbit naïve serum (**Figure 4B**). Viral titers in tail are similar between antibody treated and non-treated animals (**Figure 4C**), but the former had lower virus titer in ovaries compared to non-treated animals (**Figure 4D**), indicating that the antibody response seems to be efficient in reduce viral dissemination to internal organs. To further understand the role of antibodies in this model and to circumvent issues associate with an interspecific serum transfer [34], animals deficient in B-cells (µMT) were infected with VACV-WR as described before. µMT mice did not show any sign of generalized disease and did not die upon tail scarification infection with VACV-WR (**Figure 5A and 5B**). These animals harbored low levels of viral replication in ovaries at days 10, 15 and 28 p.i. It corroborates the hypothesis that the antibodies might be responsible for the control of viral dissemination. By day 60 p.i., all µMT animals have completely cleared the infection (**Figure 5D**). This kind of persistent infection was also seen in other viral infections in µMT animals [35]. Previous studies had demonstrated the importance of the B cell response to the control of poxvirus infection. Upon intraperitoneal infection with VACV-WR, animals that are unable to mount a humoral response had signs of severe disease with consequent death [15]. Previous studies had demonstrated that infection of µMT mice with the exclusive mouse pathogen *Ectromelia virus* (ECTV) led to virus persistence in internal organs and host death late in infection [8], [16]. The difference between the outcomes seen in these studies and the one presented here is likely due to different models of infections and viruses used, respectively [36]. Moreover, PBMC from human donors infected with wild strains of VACV showed a remarkable down-modulation of monocytes, CD4^+^ T cells and B cells (Gomes *et al*., unpublished results), highlighting the importance of these cell types in the immune response to VACV.

The next step was to evaluate the contribution of T cells to VACV immunity using the T-cell deficient mice (NUDE athymic, *nu/nu*) and their normal littermates (*nu/+*). The NUDE athymic animals have a defect in thymus' development and do not posses mature T cells [37]. These animals had a very similar outcome of infection as *Rag1* ^−/−^, with severe weight loss and 100% mortality (**Figure 6B**). It is worth to mention that the median survival was the same in NUDE athymic and *Rag1* ^−/−^ mice (17 days) (**Figure 2B** **and** **6B**). Viral titers were also significantly higher in tails of NUDE mice compared to WT animals (**Figure 6C**). Like C57BL/6 animals, normal *nu/+* mice did not harbor viral replication in internal organs at any time analyzed, contrasting with NUDE animals, which had high viral titers in spleen (**Figure 6C**). Another study showed that NUDE athymic mice also succumb to a localized infection with VACV strain Lister, reinforcing the role of T cells in the immune response to VACV [38]. Despite the compelling evidence for the requirement of T cells in the control of primary VACV presented here, we could not rule out the role of the antibodies, once NUDE animals had significantly lower IgG titers compared to *nu/+* mice (**Figure 6D**). This T helper-dependent production of antibodies is well known in cases of poxvirus infection in humans [39] and in mice [15].

To further confirm the role of T cells in this model, *Rag1* ^−/−^ animals were passively transferred intravenously with C57BL/6 spleen-derived populations enriched in CD4^+^ or CD8^+^ T cells or both cells in combination. Animals passively transferred with both cell types were fully protected against morbidity (**Figure 7A**) and mortality (**Figure 7B**), while animals that received only one cell type respond poorly to infection, with a significant weight loss between days 10 and 15 p.i. (**Figure 7A**). The survival curve of all reconstituted animals were significant different from *Rag1* ^−/−^ non-treated mice (**p<0.01, **Figure 7B**), but fully protection was achieved only in the presence of both cell types. Control animals that received WT whole splenocytes did not show any signs of generalized disease (**Figure 7A** and data not shown). All animals that were passively transferred showed a remarkable reduction in viral replication in tail and ovaries, and this reduction was more pronounced in animals that received both T cells (**Figure 7C**). In agreement, lesion healing was similar between *Rag1* ^−/−^ reconstituted with WT T cells or WT whole splenocytes, and it was deficient in animals that received only one T cell subtype (**Figure S2**). The morbidity/mortality observed in animals reconstituted with CD4^+^ or CD8^+^ T cells alone was not due to a functional defect of these cells, since freshly isolated splenocytes of these mice showed a similar percentage of IFN-γ positive cells compared to the WT T cells reconstituted group (**Figure S3B and S3C**). The groups that received either T cell types or only one cell type did not show any neutralizing activity in serum, indicating that the cell population inoculated did not contain B cells, which would affect the result obtained. These findings strongly suggested that both CD4^+^ and CD8^+^ T cells are required to prevent morbidity/mortality upon primary VACV infection. The role played by host T cells in VACV localized infection seems to be the control of viral replication at the initial site, once *Rag1* ^−/−^ had higher titers and delayed healing in the tail when compared to WT mice (**Figure 2C** **and** **3B**) and the passive transfer of WT cells to *Rag1* ^−/−^ mice decrease viral replication at this site and led to complete healing, similar to animals reconstituted with whole splenocytes (**Figure 7C and S** **2**).

Several animal models and studies with human subjects have shown the relevance of T cells to the primary immune response to poxviruses [8], [11], as well as in secondary responses [25]. Our results also showed that reconstitution of *Rag1* ^−/−^ mice with CD8^+^ T cells proved more efficient than reconstitution with CD4^+^ T cells alone (**Figure 7**). Recently, Xu and coworkers reported that direct antigen presentation to CD8^+^ T cells is sufficient to prime an efficient anti-viral cytotoxic response [40], which would enable these cells to fully activate upon VACV infection in the absence of T cell helper. Another study by Spriggs and co-workers, however, reported that β2-microglobulin deficient mice, which do not have mature CD4^-^CD8^+^ T cells, did not show any signs of generalized disease when inoculated with the same dose of VACV-WR by tail scarification [22]. This discrepancy between the results could be due to the different strategies employed. While Spriggs' group used a transgenic knockout mice, we used a passive transfer strategy in a severely immunocompromised mice (*Rag1* ^−/−^). It has been demonstrated that, in the absence of B cell/antibody response (as in our strategy), CD8^+^ T cells can protect mice from an otherwise lethal infection with VACV [6], [15]. The role of CD8^+^ T cells in this model deserves future detailed studies.

As far as we concern, this is the first study to use a systematic approach to access immunity to VACV using the tail scarification model in mice, which more close resembles the vaccination by skin punctures in humans [10]. Taken together, our results strongly suggest that both T and B cells are required to VACV clearance in mice. Based on the findings presented, we hypothesize the following scenario: upon VACV infection by tail scarification, T cells are recruited to the initial site where they control viral replication (**Figure 2C** **and** **6C**). The activation and attraction of T cells to the site of infection is important not only for the control of viral replication (**Figures 2** **and** **6**), but also for the priming of B cells with the consequent production of anti-VACV antibodies [33], [39](**Figure 6D**). The anti-VACV antibodies are then important to control viral dissemination to internal organs in the late time of infection (**Figures 4D** **and** **5D**). The complete understand of the immune mechanisms responsible for protection of a primary poxvirus infection is of major interest due to the serious adverse events post smallpox vaccination. This knowledge will enable us to better identify individuals with higher risk of complications after vaccination or natural infection with poxvirus, design more efficient poxvirus-based vaccines that specific stimulate the T cell response and design therapeutic strategies for treatment of poxvirus infections in humans.

## Materials and Methods

### Ethics Statement

All experimental protocols were carried out in strict accordance with the recommendations in the Guide for the Care and Use of Laboratory Animals of the National Institutes of Health. The protocol was approved by the Ethics Committee for Animal Experimentation (CETEA/UFMG, Brazil, protocol #110/2008) and the CDC Institutional Animal Care and Use Committee (IACUC, USA, protocol #1781DAMMOUC-A3). Animals that showed any signs of pain and distress were treated with the analgesic buprenorphine (0.05–0.1 mg/kg) given subcutaneously every 8–12 hours in an effort to minimize suffering.

### Virus and Cells

*Cercopithecus aethiops* epithelial kidney cells (BSC-40, ATCC number CRL-2761) were maintained in RPMI 1640 medium (Gibco, USA) supplemented with 10% fetal calf serum (FCS, Gibco, USA) and penicillin (200 U/ml), gentamicin (50 mg/ml) and fungizone (2.5 mg/ml). The VACV strain Western Reserve (WR, ATCC number VR-1354) was propagated in BSC-40 cells and purified using ultracentrifugation in sucrose gradient.

### Mice and Infection

C57BL/6 mice were provided by CEBIO/UFMG, Brazil. *Rag1^−/−^* and IFNγ^−/−^ animals were provided by René Rachou Institute (FIOCRUZ, Brazil). IL-12/23 knockout (IL12/23p40^−/−^), tumor necrosis factor receptor knockout (TNFrp55^−/−^) and NUDE athymic mice (*nu/nu*) and their wild type counterpart (*nu/+*) were provided by the Biochemistry and Immunology Department/UFMG, Brazil. IL-12/23p40^−/−^ matrices were a kind gift from Dr. Luiz Vicente Rizzo (Universidade de São Paulo, São Paulo, SP, Brazil), TNFrp55^−/−^ were purchased from Jackson Laboratory (B6.129-Tnfrsf1a^tm1Mak^/J, Bar Harbor, MN, USA). Additionally, C57BL/6 (C57BL/6J), Rag1^−/−^ (B6.129S7-Rag1tm1Mom/J), IFNγ^−/−^ (B6.129S7-Ifngtm1Ts/J), iNOS^−/−^ (B6.129P2-Nos2tm1Lau/J) and µMT (B6.129S2-Igh-6tm1Cgn/J) mice were purchased from the Jackson Laboratory. Food and water were provided *ad libitum* and the animals were used between 8 and 12 weeks of age. Animals were anesthetized with an intraperitoneal injection of Ketamine (100 mg/Kg of body weight) and Xylazine (10 mg/Kg) diluted in sterile phosphate buffered saline (PBS). Thirty scarifications were made with a 26 G syringe in 1 cm along the base of the tail avoiding bleeding, as previously described [10]. After that, 10 µl of PBS containing 10^7^ plaque forming units (p.f.u.) of VACV-WR were added to the area and allow to air dry. Only PBS was added in the mock-infected animals. All animals were daily weighted and checked for signals of disseminated disease (such as ruffling fur, arched back, and decreasing activities). Any animal that presented more than 25% of weight loss was humanely euthanized by cardiac puncture followed by cervical dislocation, both performed under deep anesthesia.

### Viral Titration in Organs

At different times post-infection, animals were humanely euthanized as described above and tail, spleen and ovaries were aseptically removed, weighted, and stored at −80°C until use. The samples were homogenized in 1 ml of PBS using a 2000Geno/Grinder (SPEX CertiPrep, USA). The homogenates were sonicated for three minutes at 40% amplitude, freeze and thawed twice (−80°/37°C), sonicated again with the same conditions and then serially diluted in RPMI 2% fetal calf serum (FCS). The dilutions were added to monolayers of BSC-40 cells seeded in 6-well plates, incubated for one hour at 37°C and 5% CO_2_ atmosphere and then 2 ml of the medium were added to each well and further incubated at the same conditions for 48 hours. After that time, cells were stained with a crystal violet solution (0.5% crystal violet, 10% ethanol and 1% paraformaldehyde) for 20 minutes, washed again and the viral plaques were counted. The number of plaques was multiplied by the reciprocal of sample dilution and converted to p.f.u./g.

### ELISA

Purified aliquots of VACV-WR were diluted in PBS and 10^6^ p.f.u. were added per well in a 96-well plate (NUNC Immunolon Maxisorb). The plates were incubated overnight at 4°C, washed three times with PBS containing 0.05% Tween-20 (PBS-T) and then blocked for two hours at 37°C with 250 µL of PBS-T containing 1% bovine serum albumin (BSA). The plates were washed again and then 100 µL of the serum serially diluted in a 1∶2 ratio in PBS-T 0.5% BSA were added per well. After 90 minutes at 37°C, the plates were washed again and 100 µL of horseradish peroxidase-conjugate anti mouse-IgG (KPL, Maryland, USA) diluted 1∶1.000 in PBS-T were added per well and incubated at 37°C for 60 minutes. Following this, the plates were washed five times with PBS-T and the substrate (citrate buffer 0.1 M pH 5.0, containing 0.3 mg/ml of ortho-phenylenediamine (OPD) and 0.03% hydrogen peroxide) was added and incubated in dark for 5 to 15 minutes. The reaction was stopped by addition of 40 µl per well of sulfuric acid 2N and the optical densities were measured at 492 nm (Stat Fax 2100 Microplate Reader, Awareness Technology, USA). The VACV specific IgG titer was given as the highest serum dilution that exhibited an absorbance above the median plus two standard deviations of five mock-infected animals, as previously described [41], [42].

### Plaque Neutralization Reduction Test (PRNT)

Sera samples were heat-inactivated at 56°C for 30 minutes, serially diluted in a 1∶2 ratio in RPMI 1640 with 2% FCS and incubated at 37°C for one hour with the same volume of RPMI 2%FCS containing 10^3^ p.f.u./ml of VACV-WR. At the same time, the viral suspension was also incubated with a 1∶20 dilution of FCS in RPMI to serve as a virus control. After that, 400 µl of this mixture was added to BSC-40 cells seeded in 6-wells plates, incubated for one hour at 37°C and 5% CO_2_ atmosphere and then 2 ml of the medium were added to each well and further incubated at the same conditions for 48 hours. The cells were then stained with a solution of crystal violet for 20 minutes and the viral plaques were counted. The results are expressed as the highest serum dilution that was able to neutralize at least 50% of viral plaques (PRNT_50_).

### Passive transfer of anti-vaccinia serum and naïve T cells

For immune serum transfer experiments, 500 µl of anti-vaccinia rabbit serum (PRNT_50_ titer: 8,000 neutralizing units per ml) was administered intraperitoneally at day 6 p.i. and 250 µl was intraperitoneally administered weekly thereafter. For control groups, the same regimen was applied using naïve rabbit serum.

For passive transfer of T cells, spleens of naïve C57BL/6 mice were aseptically removed and made into single-cell suspensions. Following osmotic lysis of red blood cells with 1X RBC Lysis Buffer (eBioscence, USA), splenocytes were washed and the population was enriched in CD4^+^ and CD8^+^ T cells using the Dynal® Mouse CD4 Negative Isolation Kit and Dynal® Mouse CD8 Negative Isolation Kit respectively (Invitrogen Dynal AS, Oslo, Norway), according to manufacturer's instruction. These kits are based on negative selection that produces antibody and magnetic beads-free cells for posterior use. Viable cells were identified by exclusion of 0.4% Trypan Blue (Gibco, USA), washed twice in PBS and injected into the tail vein of *Rag1* ^−/−^ mice. The first group received 5×10^5^ CD4+ T cells and 5×10^5^ CD8+ T cells, the second only 10^6^ CD4+ T cells and the third only 10^6^ CD8+ T cells. The isolation purity was confirmed by flow cytometry. As a positive control, *Rag1* ^−/−^ mice were reconstituted with 10^7^ total splenocytes from naïve C57BL/6 mice prepared as describe above. Four days after inoculation, these animals were infected as described earlier.

### Flow Cytometry

For flow cytometry, spleens of mice were pooled, made into single-cell suspensions, counted and the viability analysis with Trypan Blue was performed. After that, 10^6^ splenocytes were incubated with Mouse BD Fc Block (BD Pharmingen, USA) for 10 minutes on ice. The cells were then stained for cell surface molecules, fixed, permeabilized, and stained for intracellular molecules using the Cytofix/Cytoperm kit (BD Biosciences, USA) according to the manufacturer's instructions. The antibodies used were PE-Hamster Anti-Mouse CD3e (clone 145-2C11) (BD Pharmingen, USA), PerCP-Rat Anti-Mouse CD8a (clone 53-6.7) (BD Pharmingen, USA), APC- Rat Anti-Mouse CD4 (clone RM4-5) (Invitrogen, USA), FITC-Rat Anti-Mouse IFN-γ (clone XMG1.2) (Invitrogen, USA), besides the appropriate isotypes controls. One hundred thousand events were captured with FACSCalibur machine and the results analyzed using the FlowJo software.

### Statistical analysis

Viral titers, ELISA IgG titers and weight curves were compared using Student t test or one-way ANOVA test. Survival curves were compared with log-rank test. All statistical analyses were performed using the GraphPad Prism 4 software (La Jolla, CA, USA).

## Supporting Information

Figure S1 **IFNγ deficient animals had slightly different lesion kinetic compared to WT mice.** C57BL/6 animals and IFNγ and iNOS deficient animals were anesthetized and infected with 10^7^ p.f.u. of VACV-WR by tail scarification and the site of inoculation photographed at days 0, 5, 10 and 15 post-infection. IFNγ deficient animals presented a more purulent lesion with poor healing throughout the experiment. This phenotype is not associated with the lack of induction of iNOS, once iNOS deficient animals showed the same lesion kinetic as C57BL/6 mice. Animals mock-infected displayed the same macroscopic appearance at the site regardless the strain analyzed. In this figure, only WT mock-infected animals are shown. All the photos shown are representative of the group.
(TIF)

Figure S2 **Lesion kinetic in** ***Rag1*** **^−/−^ animals passively transferred with WT T cells.** *Rag1* ^−/−^ mice were passively transferred with 5×10^5^ CD4^+^ plus 5×10^5^ CD8^+^ T cells (WT T cells), or 10^6^ CD4^+^ T cells or 10^6^ CD8^+^ T cells from WT (C57BL/6) mice. All animals were infected four days later and followed until day 60 p.i. As a control, *Rag1* ^−/−^ received also 10^7^ splenocytes of C57BL/6 (splenocytes). The inoculation site was photographed at days 0, 5, 10 and 15 p.i. The photos shown are representative of the group. The lesion kinetic in *Rag1* ^−/−^ mice is shown to comparison purposes.
(TIF)

Figure S3 **Functionality of splenocytes after passive transfer of WT T cells to** ***Rag1*** ^−/−^ **mice. (A)** Flow cytometric analysis of the T cell-enriched population (CD3^+^CD4^+^ to the left and CD3^+^CD8^+^ to the right) derived from spleens of C57Bl/6 mice. Numbers shown are percentage of total cells expressing both markers. **(B)** *Rag1* ^−/−^ mice were passively transferred with different combination of T cells from WT (C57Bl/6) mice. At days 10 and 15 p.i., spleens of these mice were pooled, made into single-cell suspension and stained for intracellular IFN-γ. Data are shown as histograms. Gray-filled histograms represent isotype controls. **(C)** The percentage of IFN-γ^+^ splenocytes in the different groups is shown graphically.
(TIF)
