# Supplementary figures and images for: Adverse Events Post Smallpox-Vaccination: Insights from Tail Scarification Infection in Mice with Vaccinia virus
Source: PLoS One. 2011 Apr 15;6(4):e18924. doi: 10.1371/journal.pone.0018924 (PMC3078145; doi:10.1371/journal.pone.0018924)

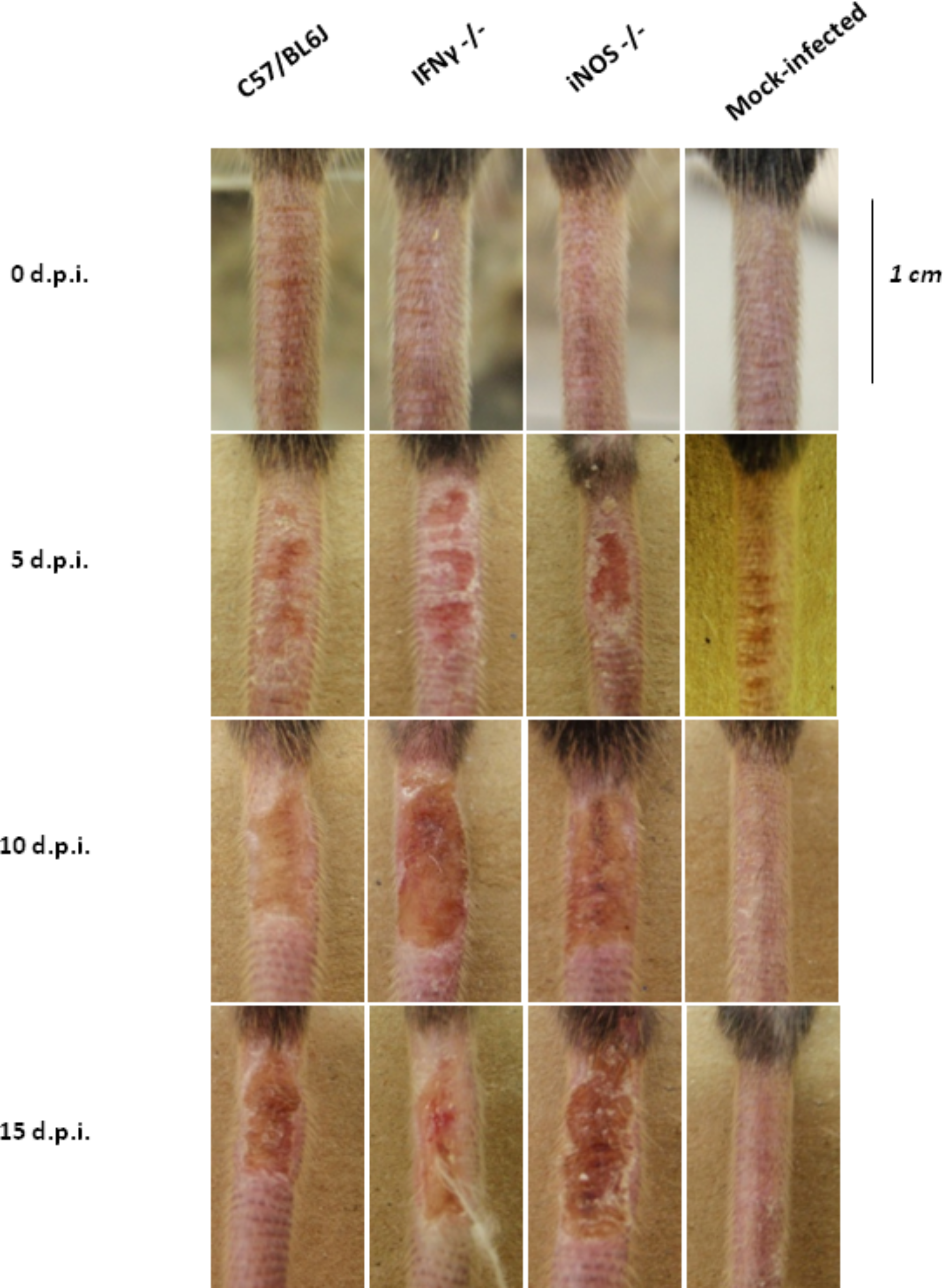

Supplement: Figure S1 — IFNγ deficient animals had slightly different lesion kinetic compared to WT mice. C57BL/6 animals and IFNγ and iNOS deficient animals were anesthetized and infected with 107 p.f.u. of VACV-WR by tail scarification and the site of inoculation photographed at days 0, 5, 10 and 15 post-infection. IFNγ deficient animals presented a more purulent lesion with poor healing throughout the experiment. This phenotype is not associated with the lack of induction of iNOS, once iNOS deficient animals showed the same lesion kinetic as C57BL/6 mice. Animals mock-infected displayed the same macroscopic appearance at the site regardless the strain analyzed. In this figure, only WT mock-infected animals are shown. All the photos shown are representative of the group. (TIF) [file pone.0018924.s001.tif]

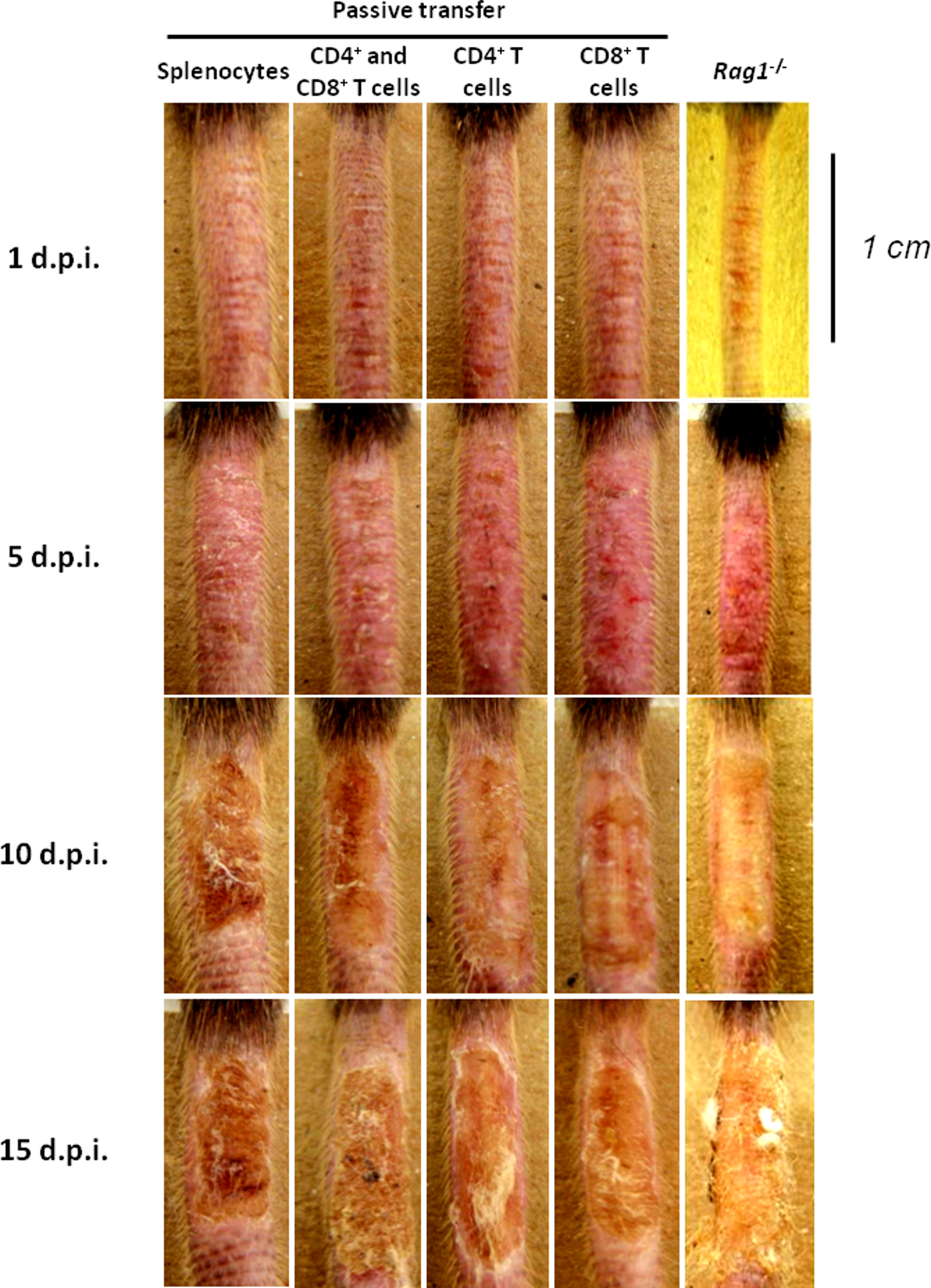

Supplement: Figure S2 — Lesion kinetic in Rag1 −/− animals passively transferred with WT T cells. Rag1 −/− mice were passively transferred with 5×10^5^ CD4+ plus 5×10^5^ CD8+ T cells (WT T cells), or 106 CD4+ T cells or 106 CD8+ T cells from WT (C57BL/6) mice. All animals were infected four days later and followed until day 60 p.i. As a control, Rag1 −/− received also 107 splenocytes of C57BL/6 (splenocytes). The inoculation site was photographed at days 0, 5, 10 and 15 p.i. The photos shown are representative of the group. The lesion kinetic in Rag1 −/− mice is shown to comparison purposes. (TIF) [file pone.0018924.s002.tif]

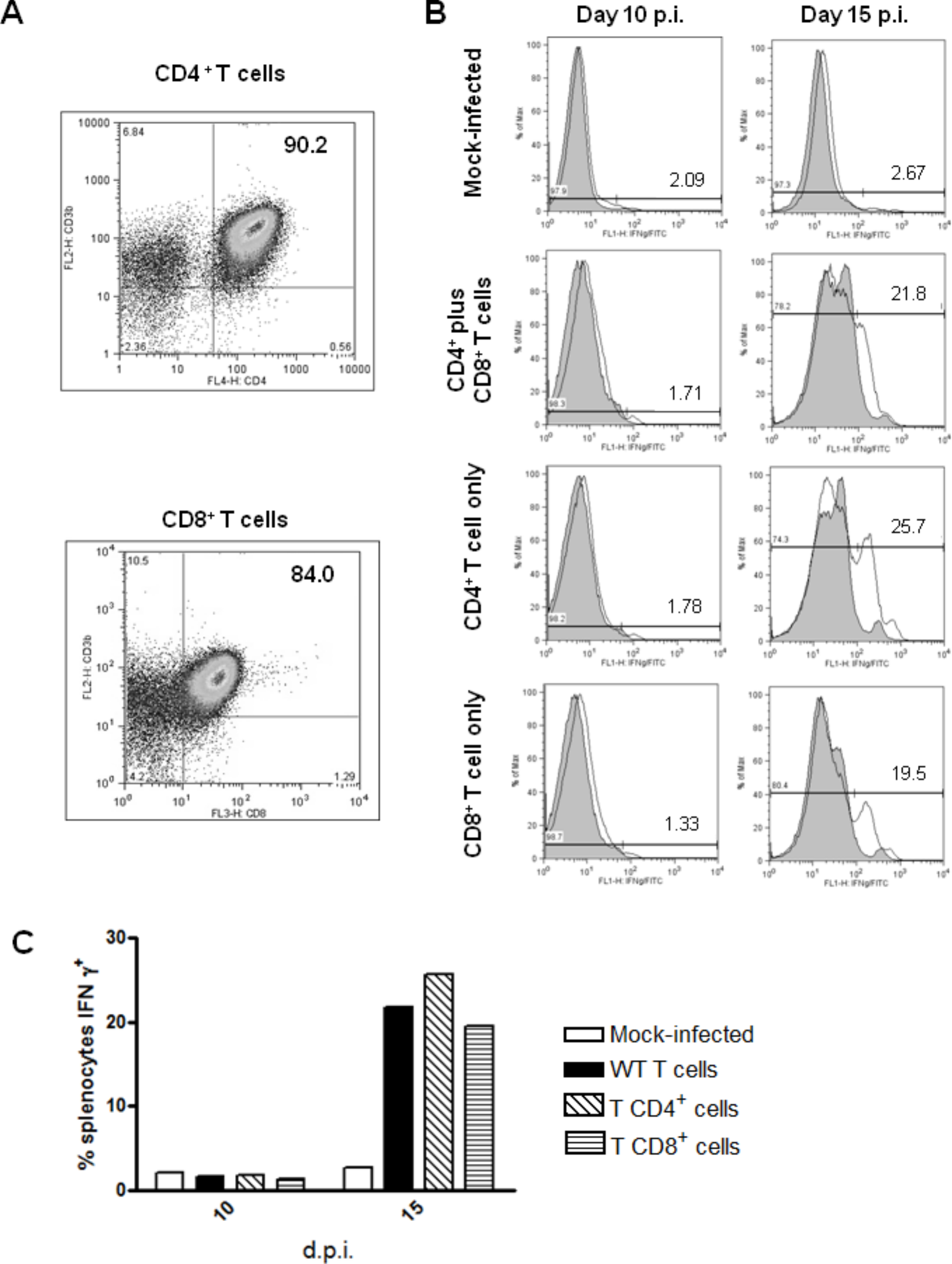

Supplement: Figure S3 — Functionality of splenocytes after passive transfer of WT T cells to Rag1 −/− mice. (A) Flow cytometric analysis of the T cell-enriched population (CD3+CD4+ to the left and CD3+CD8+ to the right) derived from spleens of C57Bl/6 mice. Numbers shown are percentage of total cells expressing both markers. (B) Rag1 −/− mice were passively transferred with different combination of T cells from WT (C57Bl/6) mice. At days 10 and 15 p.i., spleens of these mice were pooled, made into single-cell suspension and stained for intracellular IFN-γ. Data are shown as histograms. Gray-filled histograms represent isotype controls. (C) The percentage of IFN-γ+ splenocytes in the different groups is shown graphically. (TIF) [file pone.0018924.s003.tif]
